# New Appliances and Things Medical

**Published:** 1902-05-10

**Authors:** 


### NEW APPLIANCES AND THINGS MEDICAL.

[We shall be glad to receive at our Office, 28 & 29 Southampton Street, Strand, London, W.C., from the manufacturers, spec ens of all new preparations and appliances which may be brought out from time to time.]

### BOTTLES AND CORKS.

(AYRTON AND SAUNDERS, 34 HANOVER STREET, LIVERPOOL.)

MEDICAL men who do their own dispensing, and the authorities of public institutions who require bottles and corks for this purpose, will find it both economical and advantageous to deal directly with the large wholesale firm of Ayrton and Saunders. As an example of the moderate prices at which their goods are listed we quote the price of their Faro, Lisbon, and Cadiz corks which are in the case of the two first mentioned 7d. per gross, and in that of the last 1s. per gross. We have examined these corks and find them carefully selected, close-grained and firm, and well suited for the purpose of dispensing.

### ROENTGEN RAY PHOTOGRAPHY.

(W. H. BAILEY AND SON, 38 OXFORD STREET, LONDON, W.)

FOR the convenience of their customers, W. H. Bailey and Son, of 38 Oxford Street, have established a complete X-ray apparatus in a room on their own premises. The apparatus consists of the most modern and approved form of battery and induction coil, the latter capable of giving a 10-inch spark. The apparatus which is under the charge of a resident expert is capable of doing the most delicate work in X-ray photography for the detection of fractures, dislocations, and the discovery of foreign bodies. Medical men are invited to send their patients to 38 Oxford Street, or arrangements can be made for attending patients at their own houses.

### HORN'S ANTI-MICROBE POCKET-INHALANT.

(GLENDOWER, BOURNEMOUTH WEST.)

THE pocket-inhaler which is referred to above is an unbreakable, unspillable, and convenient capsule for carrying in the pocket. It is so constructed that it can be easily opened at one end, at which a small plug of cotton-wool is inserted, and into this cotton-wool a few drops of the antiseptic and deodorising fluid are poured. The fluid is described as a terpeneless extract of pine-needles, eucalyptus, and thyme. When inhaled by the nose it has a pleasant, healthy odour, and as would be expected from the well-known therapeutic properties of the constituent oils, it is highly efficacious in many pulmonary and throat affections. The apparatus is a useful means of applying antiseptic vapours to the nasal mucous membrane. The price of each inhaler complete with the medicament for charging is 2s. 6d.

